# Colour Polymorphism Protects Prey Individuals and Populations Against Predation

**DOI:** 10.1038/srep22122

**Published:** 2016-02-23

**Authors:** Einat Karpestam, Sami Merilaita, Anders Forsman

**Affiliations:** 1 Ecology and Evolution in Microbial Model Systems, EEMIS, Department of Biology and Environmental Science, Linnaeus University, SE-391 82 Kalmar, Sweden; 2 Behavioural and Evolutionary Ecology Group, Environmental and Marine Biology, Department of Biosciences, Åbo Akademi University, FI-20520 Turku, Finland

## Abstract

Colour pattern polymorphism in animals can influence and be influenced by interactions between predators and prey. However, few studies have examined whether polymorphism is adaptive, and there is no evidence that the co-occurrence of two or more natural prey colour variants can increase survival of populations. Here we show that visual predators that exploit polymorphic prey suffer from reduced performance, and further provide rare evidence in support of the hypothesis that prey colour polymorphism may afford protection against predators for both individuals and populations. This protective effect provides a probable explanation for the longstanding, evolutionary puzzle of the existence of colour polymorphisms. We also propose that this protective effect can provide an adaptive explanation for search image formation in predators rather than search image formation explaining polymorphism.

Colour polymorphism is the coexistence within a population of two or more discrete, genetically influenced colour pattern variants^1^. This widespread phenomenon has long puzzled ecologists and evolutionary biologists^2^,^3^,^4^,^5^,^6^,^7^,^8^,^9^,^10^,^11^,^12^,^13^,^14^. Much of the research on polymorphism has aimed to identify the processes and mechanisms which might contribute to the maintenance of multiple colour variants that are not selectively neutral^2^,^6^,^11^,^15^,^16^,^17^. In the case of colour polymorphism in animals, major lines of investigation have concerned the roles of predator-prey interactions. It has been suggested that predators that exploit polymorphic prey may suffer from reduced performance compared with predators that exploit monomorphic populations, owing to perceptual, behavioural and ecological mechanisms^10^,^18^,^19^,^20^,^21^,^22^,^23^,^24^,^25^.

A long-standing controversial question that has not yet been resolved concerns the possible adaptive value of polymorphisms and whether they can increase the fitness of populations^2^,^6^,^13^,^26^,^27^,^28^,^29^,^30^. That colour polymorphism may reduce predation was proposed a long time ago^4^,^5^, but surprisingly few attempts have been made to experimentally evaluate this hypothesis. One reason is that the critique and misunderstandings regarding group selection (reviewed in^31^) may have discouraged students and hampered progress in this research area. It is also empirically challenging to evaluate the question whether polymorphism is adaptive because the proposed protective effect involves mechanisms at multiple levels: the aforementioned consequences of prey polymorphism for foraging predators; the consequences of predation for prey individuals that vary in colour pattern; and the consequences of predation for populations of prey that vary in the level of polymorphism they exhibit.

Experimental approaches using artificial or semi-artificial systems have provided useful solutions and important insights in the above issues. However, few experimental studies have been carried out in such a way that the results allow for evaluating the hypothesis^4^,^5^ that colour polymorphism protects individuals and populations from predation (summarised in Table 1 and Supplementary Information). A key message that emerges from a review of these studies is that in most previous experiments prey were presented in sequences^10^,^32^,^33^ rather than in groups^18^,^34^,^35^. The effects of polymorphism on predator efficiency and prey vulnerability may differ if predators instead encounter aggregations with multiple potential prey individuals. In many cases it would be realistic to hypothesize that subsequent encounters of prey would be connected to each other in time and place.

Another key message that emerges from the review of previous work (Table 1, Supplementary Information) is that all but one^35^ of the six previous experiments the protective polymorphism hypothesis was evaluated based on comparisons of fitness (rate of or time to detection) for individuals in polymorphic versus monomorphic treatments. We are aware of only one study that has tested whether polymorphism increases the survival of populations. Wennersten and Forsman^35^ used artificial prey made of dyed pastry in a field experiment and compared survival of polymorphic and monomorphic groups that were exposed to predation by wild birds. In their experiment, coloration affected survival of both individuals and populations, but polymorphic populations that included prey of four different colours did not go extinct at a lower overall rate than monomorphic populations^35^. The colours of prey used in that experiment were not concealing; it has been hypothesized that the protective effect of polymorphism should be greater when prey are more difficult to detect^9^,^33^,^34^.

As the review of previous work indicates, it remains an open question whether natural colour polymorphic variation can afford protection from predation to the extent that it increases survival of prey populations. Furthermore, only one study^33^ has addressed the question whether the protective effect of polymorphism increases monotonically with increasing levels of polymorphism, follows the law of diminishing returns, or peaks at some intermediate level of polymorphism. It is necessary to answer the question how the number of alternative morphs influences the outcome of predator-prey interactions because this can affect evolutionary transitions from mono- to polymorphism and the level of diversity in polymorphic populations. Little is also known about how the consequences of being member of a polymorphic group, in terms of detection, depend on colour pattern. Yet, any morph specific effects may impact on individual decision making and affect the size and composition of animal aggregations.

Here we set out to shed new light on these unresolved issues regarding how variable colour patterns influence predation risk. We conducted a computer-based detection experiment that was designed to investigate: (i) whether and how polymorphism of natural camouflaged colour patterns affects survival of individuals and of populations; (ii) the shape of the relationship linking survival to the level of polymorphism; and (iii) whether the protective effect of polymorphism is similar or different for alternative morphs.

## Results

**Figure 1:**
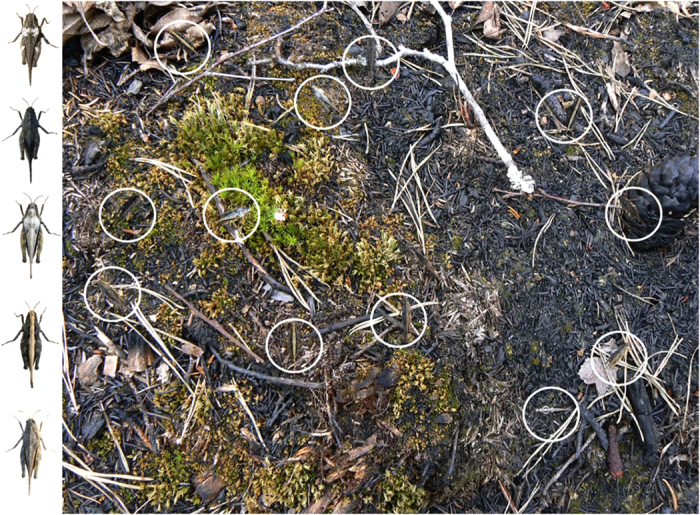
Prey and visual background used for the detection experiment. (Left) The five colour morphs of *Tetrix subulata* grasshoppers used as prey. From top to bottom: transversal bar, black, grey, striped and brown. Photos by Einat Karpestam. (**Right)** An example of tetramorphic image used in the detection experiment. The 12 white circles have been added afterwards to indicate the location of the grasshoppers in the image. This image represents a tetramorphic population consisting of the grey, black, striped and brown colour morphs. Grasshopper size was scaled to 12 mm and was in proportion to the distance from which the background image was photographed. Photos by Einat Karpestam.

For this study, we used photographs of the colour polymorphic grasshopper *Tetrix subulata* implemented in images of their natural background (Fig. 1). These images were displayed on computer screens in groups that represented four levels of polymorphism, and presented to humans that acted as visual predators^10^,^36^,^37^. A total of 3360 grasshopper images were presented in 280 populations of 12 prey individuals each, representing four levels of polymorphism (1, 2, 3 or 4 morphs). The 70 human subjects detected the majority (70%) of grasshoppers, but only 14% of the populations went ‘extinct’.

### Survival of individuals

**Figure 2:**
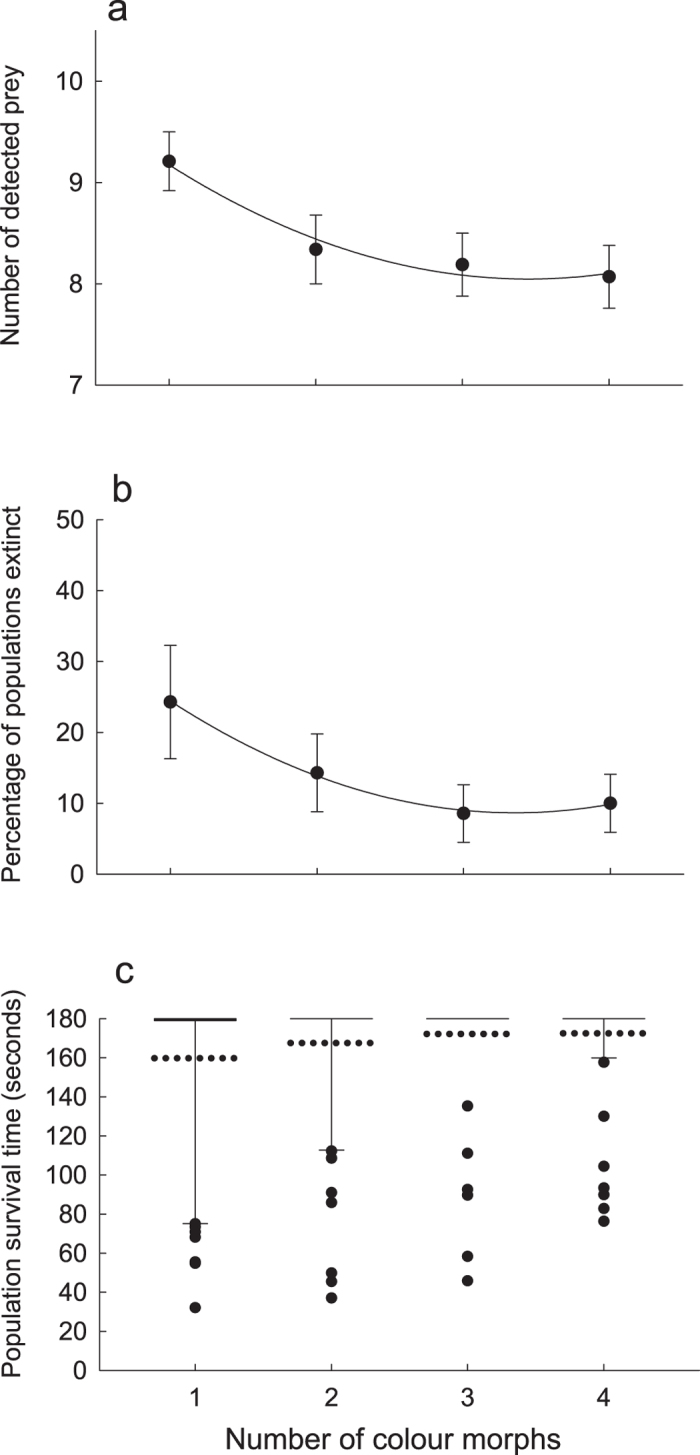
Effects of colour polymorphism on prey detection. Comparisons of (**a**) number (mean +/− se) of detected grasshoppers; (**b**) percentage of populations that went extinct (error bars denote 90% confidence intervals); and (**c**) population persistence time (box and whisker plot) among prey presentation treatments with different levels of colour pattern polymorphism (number of colour morphs included in the population). In the bottom box and whisker plot panel, the solid lines (at 180 seconds) indicate medians as well as 25th and 75th percentiles, dotted lines indicate means, whiskers below indicate 10th percentiles, and dots indicate outlying observations. Data on population survival time was strongly skewed because the majority of populations survived for 180 seconds; all medians, and the upper and lower quartiles were therefore 180 sec.

The number of different morphs included in the population had strong effect on survival of individuals of that population (survival analysis, Mantel–Cox χ^2^ = 22.85, *df* = 3, *P* < 0.0001; Log-rank test for trend, χ^2^ = 16.89, *df* = 1, *P* < 0.0001). Number of detected grasshopper images decreased in a curvilinear decaying manner with increasing polymorphism level (mixed-effects model analysis implemented using procedure MIXED in SAS, random effect of subject: estimate = 1.07 ± 0.045 SE, *Z* = 2.38, *P* = 0.0086; χ^2^ = 8.7, *df* = 1, *P* < 0.005; fixed linear effect of polymorphism level: *F*_1,209_ = 7.86, *P* = 0.0055; fixed curvilinear (quadratic) effect of polymorphism level: *F*_1,209_ = 6.34, *P* = 0.0125; Fig. 2a).

### Percentage of populations that went extinct

The proportion of prey populations that went extinct decreased in a curvilinear decaying manner with increasing levels of polymorphism (GLMM, linear effect of polymorphism level: *F*_1,278_ = 6.57, *P* = 0.0109; curvilinear quadratic effect of polymorphism level: *F*_1,278_ = 5.22, *P* = 0.023; random effect of subject: estimate = 0.43 ± 0.37 SE; Fig. 2b).

### Persistence time of populations

Time to population extinction (persistence time) varied among treatments and increased significantly with increasing polymorphism level (ordered heterogeneity test, *p*_*c*_*r*_*s*_ = 0.96, *k* = 4 groups, *P* < 0.005; Fig. 2c).

### Benefits of polymorphism-relative survival of each colour morph in monomorphic versus polymorphic populations

**Figure 3:**
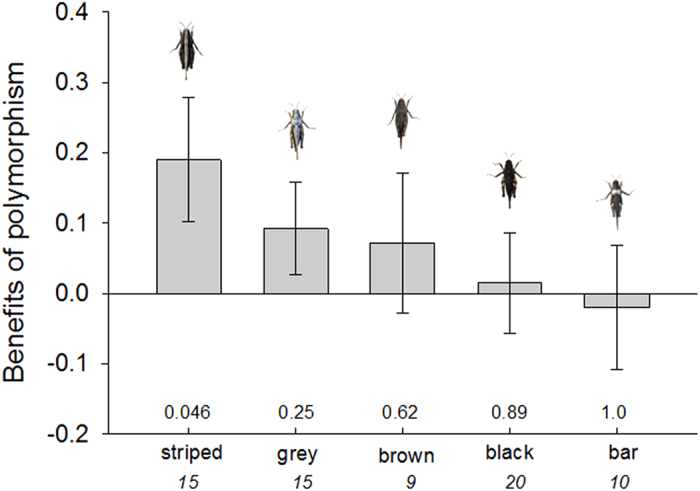
Consequences of belonging to a polymorphic prey population for prey that represent different colour morphs. Benefit of polymorphism was calculated based on paired comparisons that represent the differences (mean ± SE) between the mean proportion of *Tetrix subulata* grasshopper images representing five different colour morphs presented on computer screens in monomorphic and in polymorphic (average across populations including 2, 3 or 4 different morphs) populations that were detected by human ‘predators’. Positive values indicate that the colour morph was detected at a higher rate in monomorphic than in polymorphic populations, and negative values indicate that the colour morph was detected at a lower rate in monomorphic than in polymorphic populations. Numbers below the vertical axis indicate number of paired replicates. Numbers above the vertical axis denote *P*-values from Wilcoxon signed rank sum tests (i.e., the non-parametric version of paired *t*-test). Photos by Einat Karpestam.

The effect of experimental polymorphism level (1, 2, 3 or 4 morphs) on the proportion of presented grasshopper images of each colour morph that was detected varied among the five different morphs (GLMM, effects of the interaction between colour morph and number of morphs in the population: χ^2^ = 20.04, *df* = 12, *P* = 0.066). The model including the two-way interaction effect (Akaike Information Criterion, AIC = 230.6) was more appropriate than a model without the interaction (AIC = 232.3)^38^. The striped morph was detected at a significantly lower rate in polymorphic than in monomorphic populations (Wilcoxon signed rank sum test, *S* = 35, *n* = 15, *P* = 0.046), but the protective effect of polymorphism was weak to non-existing for the other morphs (Fig. 3).

**Figure 4:**
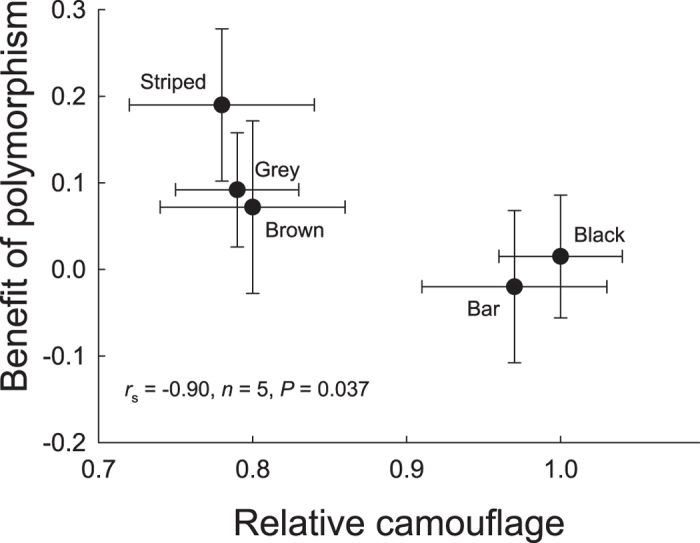
Benefit of polymorphism as a function of relative camouflage for five *Tetrix subulata* colour morphs. Benefit of polymorphism was calculated as explained in the legend to Fig. 3. Relative camouflage was based on estimates of the average proportion images of each morph that was detected in monomorphic populations and expressed relative to the proportion for the most protected morph with the lowest detection rate, which was assigned a relative camouflage of 1.0. Error bars represent ± SE.

The benefits of polymorphism for the different morphs decreased with increasing relative camouflage (*r*_s_ = −0.90, *n* = 5, *P* = 0.037; Fig. 4).

## Discussion

We investigated: (i) whether and how polymorphism affects survival of individuals and of populations; (ii) the shape of the relationship linking survival to the level of polymorphism; and (iii) whether the protective effect of polymorphism varies among alternative morphs. Our results showed that both the number of detected grasshopper images and the proportion of prey populations that went extinct decreased in a curvilinear decaying manner with increasing polymorphism level, and that time to population extinction increased with increasing number of alternative morphs included in the population. As such, our findings provide rare (Table 1) experimental evidence in support of the protective polymorphism hypothesis^4^,^5^ that phenotypic variation in colour pattern among prey individuals decreases predator attack efficiencies, which in turn can increase persistence of prey populations.

The benefits of polymorphism for individuals and populations followed the law of diminishing returns, with the increase in protection being greatest from monomorphism to dimorphism (see also^33^). A mechanistic explanation for this finding may be that the change in the amount of attention that can be expected to be allocated per prey morph is a decaying function of increasing number of morphs. The reduction in average attention is largest in the transition from mono- to dimorphism (from 100% to 50%) and gradually declines over subsequent transitions (from 50% to 33% from two to three morphs, and from 33% to 25% from three to four morphs). This pattern of diminishing returns has implications for our understanding of the evolutionary dynamics of polymorphisms, in suggesting that the protection against predators that phenotypic diversity affords may promote evolutionary transitions from mono- to dimorphism, contribute to the spread of novel phenotypes and help maintain colour polymorphisms. Based on our present results, we expect that selection pressure promoting the increase of colour pattern variation would be strongest from a monomorphic appearance to a polymorphic appearance. To evaluate this prediction, a comparative approach could be used to examine whether dimorphic colour polymorphisms are more or less widespread than higher levels of polymorphisms. We are not aware of any such investigations.

With regard to the evolution of exuberant or massive polymorphisms (*i.e*., the coexistence of large numbers of alternative morphs)^19^,^39^,^40^ this reduced predator efficiency does not seem to be a sufficient explanation. It is possible that colour-morph specific and matching microhabitat habitat choice can provide broader conditions for exuberant polymorphisms^37^. Additional mechanisms that may account for such intriguing phenomena include reduced intra-specific competition owing to resource subdivision, niche complementarity, facilitation and beneficial social interactions among individuals belonging to different phenotypes or genotypes, negative frequency-dependent selection, and environmental heterogeneity^41^,^42^,^43^,^44^.

Our findings are important within the context of individual decision making. For instance, a large number of papers have suggested that search image formation by predators may contribute to polymorphism. The protective effect of polymorphism indicated by our results offers a mechanistic explanation for *why* predators should form search images and focus on one prey type at a time^22^; as a counter-adaptation to compensate for reduced efficiency when exploiting polymorphic prey populations. This counter-adaptation of predators may in turn further promote polymorphism through frequency-dependent selection that it may cause. With regard to optimal foraging behaviour, the observation that detection rates were lower in more polymorphic populations has implications for when predators should leave a patch or switch and search for a different or additional prey types.

The existence and utility of diversity as an escape tactic is further relevant with regard to aggregation behaviour. Based on the protective effect of polymorphism indicated by our present results, it can be hypothesized that camouflaged prey should preferentially join groups in which other members have different phenotypic attributes rather than monomorphic groups. Our comparisons of relative detection rates in monomorphic versus polymorphic treatments further suggested that the consequences of being a member of a polymorphic population varied among the five morphs (Fig. 3, see also Wennersten and Forsman^35^ and Croze^18^). The benefits of polymorphism for these different morphs decreased with increasing relative camouflage (Fig. 4). This may have reflected differences in how detectability impacts on the ability of predators to develop search images and allocate attention to specific phenotypes^20^,^45^. If the costs and benefits of group-living vary among morphs, this may in turn influence decision making and impact on the composition (individual variability) of animal groups. It can also be hypothesized from the inferior survival of monomorphic compared with phenotypically diverse populations that evolutionary gains and losses of colour polymorphism should be associated with evolutionary gains and losses of group living, or with shifts in prey density and/or encounter rate. However, we are not aware of any studies that have explored these issues.

Our main result and conclusion, that colour polymorphism can protect against predation, differs from the findings of Wennersten and Forsman^35^, the only previous study we are aware of that has tested whether polymorphism influences survival of populations. Wennersten and Forsman^35^ reported that tetramorphic populations of artificial pastry prey survived less well than monomorphic green populations, and slightly (but not significantly) better than monomorphic brown, yellow and red populations. Many aspects of study design (e.g., laboratory computer-based setting *versus* field study, human subjects *versus* natural predators, images of natural camouflaged patterned prey *versus* artificial monochromatic baits, four *versus* two levels of prey colour variation) differ between our study and that of Wennersten and Forsman^35^. Yet, that survival was lower for monomorphic than for polymorphic populations in the present study but not in that of Wennersten and Forsman^35^ lends some support to the notion^9^,^33^,^34^,^46^ that the protective value of polymorphism, and the decrease in predators ability to detect variable prey, should be more pronounced for prey phenotypes that are harder to detect (but see^34^).

To use humans as substitutes for real predators in detection experiments is an increasingly used approach to study the function and evolution of protective coloration (see Fig. 1 in Karpestam *et al*.^37^). The outcome of predator-prey interactions in the wild can be influenced by variation in predator motivation (e.g., hunger levels) or in the rate or efficiency by which predators encounter, detect, recognize, attack, capture, and kill or consume the prey. Furthermore, direct observations of natural predation events are typically rare. Our computer-based approach enabled good control of prey presentations and avoided the risks that comparisons of prey survival were influenced by variation in predator motivation or encounter rate. Our approach also allowed for unprecedented levels of replication (3360 grasshopper images, in 280 populations of 12 prey each, presented to 70 human participants each of whom received 4 levels of polymorphism) for a study addressing these questions.

For increased naturalism, photographs of real grasshopper colour morphs were used as prey and photographs of their natural habitat as backgrounds. Depending on the system under investigation, using humans as predators might generate misleading results because the visual abilities of humans can differ from those of natural predators. The use of motionless prey may also influence results. The protective values of alternative colour patterns can change depending on prey activity and movement patterns^47^,^48^, and be modified by differences in microhabitat utilization^37^. However, in our system, detection rates of grasshopper colour morphs as estimated by humans with this computer-based approach have been shown to offer reliable predictors of selection imposed by natural visual predators and conform well with spatiotemporal shifts in relative frequencies of alternative morphs in natural populations^37^. Taken together, this suggests that our results regarding the protective effects of colour polymorphism for individuals and populations can inform about the adaptive significance of polymorphisms in the wild.

In conclusion, our study provides rare experimental evidence that colour polymorphism can reduce detection rate of camouflaged prey individuals and populations. These findings are consistent with the hypothesis put forward a long time ago that polymorphism may protect against predation^4^,^5^, and further lend support to the controversial suggestion that polymorphism may be adaptive for populations^2^,^6^,^13^,^26^,^27^,^28^,^29^,^30^. Examining how the benefits that polymorphism can confer in terms of reduced susceptibility to predation impacts on individual decision making, evolution of colour polymorphisms and aggregation behaviour in animals are interesting avenues for future research.

## Methods

The approach used for the computer based experiment was similar to the one used in our previous studies designed to address related questions^10^,^36^,^37^.

### Photographic sampling of visual backgrounds and grasshopper colour morphs

Photographs of *T. subulata* habitats were taken in May-July 2010 with a digital compact camera (Panasonic Lumix DMC-TZ7, 35 mm, film camera equivalent focal length was 37 mm) using ‘macro’ mode at a vertical distance of approximately 30 cm straight from above. Pictures were taken in southeast Sweden in typical *T. subulata* habitats and under variable daylight conditions at three locations that had been previously burnt in natural or managed forest fires, that were at different stages of recovery and that represented a mixture of burnt and non-burnt habitat patches in similar proportions. A full description of the methods for photography is available elsewhere^10^,^36^,^37^. Adult *T. subulata* grasshoppers were collected at the same sites where the background pictures were taken. We collected individuals that belonged to five natural colour morphs (the black, grey, striped, brown and transversal bar morph) and brought them to the laboratory at Linnaeus University, Kalmar, for photography (Fig. 1). These five morphs were chosen because they are distinct from each other and because their frequencies have been shown to vary both among populations and over time within populations^8^,^37^. The grasshoppers were individually photographed approximately seven cm from above (shortest macro distance) on a brownish cardboard in a shaded area under natural daylight conditions.

We used the software Adobe Photoshop CS4 for image processing. Grasshopper images were cut from their original background and saved on transparent backgrounds to enable their implementation in the background images. All grasshoppers were measured for body length in the photos (from head to end of pronotum) and rescaled to 12 mm on the screen (this is within the size ranges of females and males), to exclude any size-dependent bias in detectability. Images were presented on a 15′ computer screen (Fujitsu Lifebook e series, screen resolution = 1600 × 900 pixels) in a dark room as described below. Seventy people (31 males and 39 females) ranging between 20 and 65 years of age and with normal or corrected to normal vision participated in the experiment. All people participating in the experiment were unaware of the research question.

### Presentation of the grasshoppers and the detection experiment

To test how the level of colour polymorphism of a population influences visual detection rates of individuals and extinction risk of populations, we presented images with groups of 12 grasshoppers to human subjects that acted as ‘predators’ that searched visually for prey. For the production and presentation of images, we used a purpose written program in MATLAB 2011, MathWorks^36^,^37^. For each series of presented images, the program produced unique combinations of background images and images of grasshoppers implemented in the background (Fig. 1). For this the program used seven different background images of heterogeneous habitat (see above), and seven images of different individual grasshoppers of each of the five morphs (total = 35) black, grey, striped, brown, and bar (Fig. 1). The location and the rotational angle of each grasshopper were randomized for each image. We used several different images of the background and the grasshoppers and random placement of the grasshoppers to increase realism and the generality of our results. The main concern was not whether each background, morph or combination would have been presented equally many times, but to ensure that the sequences and combinations were unpredictable and that there was low risk that a subject would learn details of the visual appearance of a background during the course of the subsequent presentations.

Before each experimental session, the subject received a verbal explanation of the task and a short practice session. The practice session started with a 20-second presentation of grasshoppers in white squares on a grey background image. In this first presentation, which purpose was to familiarize the subject with the different colour morphs, there were 10 grasshoppers, two of each morph in the image. In the next presentation, five grasshoppers, (one of each colour morph) implemented in an image of the natural background was displayed to familiarize the subject with the actual search task. The subject was asked to search and click the mouse on each grasshopper that he/she found. Only five grasshoppers were presented so that the presentation would not take too long. If no grasshoppers were detected, the instructor pointed on a grasshopper to ensure a visual detection. The practice session was identical to each subject, and after the practice session the subject was left alone to carry out the experimental session.

During the experimental session, each subject was presented four images in sequence representing the different levels of polymorphism. In each presented image there were 12 grasshoppers implemented in a background image (Fig. 1). The level of polymorphism varied between the presented images but within each level the initial proportions of alternative morphs were always equal. Thus, in an image that represented a monomorphic population all 12 grasshoppers were of same morph, in a dimorphic population there were six individuals of each of the two morphs, etc. For each image, the morphs were randomly chosen among the five available morphs. The order of the four images that represented different levels of polymorphism was varied randomly among subjects to exclude any possible biases due to learning.

The subjects were not informed about the number of grasshoppers in the images, but they knew that there was more than one grasshopper. Each image was presented up to 180 sec or until participants chose to move to the next image. The subjects were instructed to search for grasshoppers in the images and, when detecting one, to immediately click the mouse on it. The program recorded the colour morph of the grasshopper and the time to its detection. At the same time, it played a beep sound and the grasshopper image disappeared from the screen making it unavailable for clicking. The program also recorded erroneous clicks if the mouse was clicked where there was no grasshopper. In each image, there was also a ‘continue’ button. By clicking the mouse on it the subject could switch to the next image even before the 180 sec had passed, in case he/she was not able to find (more) grasshoppers or thought that all were already found. After 180 seconds, the program displayed a message that search time was over, and asked the subject to press the ‘continue’ button to switch to the next image. After the fourth image was presented, the program displayed a message thanking the subject and information on the percentage of grasshoppers that had been found in each screen.

### Analyzing survival of individuals

To test the hypothesis that individual prey are better protected from predators when they occur in polymorphic populations than when they occur in monomorphic populations, we compared survival of individual grasshoppers between populations with different levels of polymorphism. We used the time to detection of each individual, and assigned the undetected (survived) individuals the maximum duration of a trial, which was 180 sec. We employed the log-rank Mantel-Cox survival analysis to evaluate the null-hypothesis that there is no variation among the treatments irrespective of any directional pattern. The rational in using this analysis is that it can take into account also censored observations of grasshopper images that were still undetected when we terminated the experiment such that the recorded survival time is an underestimate of the actual survival time. The survival analysis was also used to calculate log-rank test for trend (GraphaPad Prism 6) to evaluate the null hypothesis of no effect against the alternative hypothesis that the protective effect for individuals increased with increasing number of morphs.

The survival analyses described above do not account for repeated measures. We therefore also analysed the data using linear mixed-effects models implemented with the procedure MIXED in SAS^38^,^49^. Number of detected prey was used as the dependent variable, and polymorphism level was treated as a continuous fixed covariate (regressor). Subject was included as a random factor to statistically control for the variation among participants in their overall capacity to detect prey, and to account for any correlations (non-independence) that might have arisen because each subject was used for each of the four treatment levels. The Kenward-Roger method was used to approximate degrees of freedom^38^. Statistical significance of the random factor was assessed using the Log-likelihood ratio test with one degree of freedom per random effect^38^,^49^. To evaluate whether the relationship linking number of detected prey to the level of polymorphism was curvilinear we performed quadratic regression analysis by using the squared polymorphism level as the covariate.

### Analyzing percentage of populations that went extinct

To evaluate the hypothesis that the proportion of extinct populations increased consistently with increasing levels of polymorphism we used generalized linear mixed models. This analysis was implemented with procedure GLIMMIX in SAS. In this approach, population survival was modelled as a binary response variable using a logit link function, the squared polymorphism level was treated as a continuous fixed variable, and subject was included as a random factor to account for repeated measures of the same subject. The polymorphism level was squared to model quadratic (curvilinear) regression.

### Analyzing persistence time of populations

Experimental trials were terminated after three minutes if the subject had not detected all prey items and data on time to population extinction were therefore skewed. To evaluate the null-hypothesis of no effect against the directional (simply ordered) hypothesis that time to population extinction should increase with increasing degree of polymorphism we carried out an ordered heterogeneity test^50^. This powerful composite test statistic combines the magnitude of the observed variation in persistence time among treatments (obtained from a non-parametric Kruskal-Wallis ANOVA for effects of polymorphism level on persistence time) and a Spearman rank correlation between observed and expected rankings of treatments.

### Comparing relative survival of each colour morph in monomorphic versus polymorphic populations

To examine whether the benefit (in terms of reduced probability of being detected) of belonging to polymorphic populations is independent of or varies among colour morphs, we conducted pairwise comparisons for each colour morph of the probability of detection in monomorphic and in polymorphic populations^35^. To this end, we first computed for each subject the proportion of presented grasshopper images that was detected in the monomorphic treatment. Next, we computed, for the same subject and for the same morph as in the monomorphic presentation, the proportion of detected images of the corresponding colour morph in the bi-, tri- and tetra-morphic treatments. These data were analysed using linear mixed-effects models implemented with procedure MIXED in SAS^38^,^49^. The proportions were arc-sin square root transformed prior to analysis, and subject was included as a random factor to account for repeated measures. As explained above, morphs were randomly assigned to presentation treatments. The morph included in the monomorphic treatment therefore was not always included in all polymorphic treatments presented to the same subject. However, the MIXED procedure can handle unbalanced data, such as when repeated measures are not available for all individuals.

### Ethics statement

The experiment was performed in accordance with the established ethical guidelines of the Declaration of Helsinki, and the Swedish legislation and ethical regulations for experimental research (http://www.codex.vr.se/en/manniska5.shtml). As our research does not involve “a physical intervention affecting a person who is participating in the research, or is conducted in accordance with a method intended to physically or mentally influence a person who is participating in the research”, it does not require ethical vetting according to the Central Ethical Review Board (http://www.epn.se/en/start/) and the Swedish Act concerning the Ethical Review of Research Involving Humans (2003:460, http://www.epn.se/media/1205/the_ethical_review_act.pdf). The experimental protocol was approved by the Linnaeus University Faculty of Health and Life Sciences and the Ethical Advisory Board in Southeast Sweden. Informed consent was obtained from all subjects who volunteered to participate after they had received a description of the setup. There was no reward for those who did participate or any repercussion for those who did not want to participate in the experiment.

## Additional Information

**How to cite this article**: Karpestam, E. *et al*. Colour polymorphism protects prey individuals and populations against predation. *Sci. Rep.* **6**, 22122; doi: 10.1038/srep22122 (2016).

## Supplementary Material

Supplementary Information

## Figures and Tables

**Table 1:** Summary of experimental studies that allow for evaluating the ‘protective polymorphism hypothesis’^4,5^ that colour polymorphic variation affords protection from predation to the extent that it increases survival of individuals and populations of prey.

Study	Experimental setting	Predators	No. of predators (x trials per individual)	Prey	Prey treatments	Prey presentation	Polymorphism protects inds	Polymorphism protects pops
Pietrewicz & Kamil^32^	Laboratory experiment, prey presented on slides to birds	Blue jays (*Cyanocitta cristata*)	5 (x24)	Photographs of *Catocala* moths	2 (1 species or mixture of 2 species)	Sequence of 16	Yes	n.a.
Knill & Allen^33^	Computer-based detection experiment	Humans	80	Artificial: squares	4 (1, 2, 6 or 12)	Sequence of 40	Yes	n.a.
Karpestam *et al*.^10^	Computer-based detection experiment	Humans	30 (x2)	Photographs of *Tetrix subulata* grasshoppers	2 (single or mixture of 3 morphs)	Sequence of 10 or 12	Yes	n.a.
Croze^18^	Field experiment	Carrion crows (*Corvus corone*)	One pair (x17)	Painted mussel shells with meat rewards	2 (1 or 3 morphs)	Groups of 27	Yes*	n.a.
Glanville & Allen^34^	Computer-based detection experiment	Humans	40	Artificial, images resembling resting moths	2 (1 or 10 morphs)	Groups of high (20 prey per screen) or low (4 prey per screen) density	Yes	n.a.
Wennersten & Forsman^35^	Field experiment	Wild birds	Unknown	Artificial, dyed pastry baits resembling butterfly larvae	2 (1 or mixture of 4 colours)	Groups of 12	Yes*	No
*This study*	Computer-based detection experiment	Humans	70 (x4)	Photographs of *T. subulata* grasshoppers	4 (1, 2, 3, or 4 morphs)	Groups of 12	Yes*	Yes

n.a. denotes that it was not tested whether polymorphism influenced survival of populations (as opposed to survival of individuals).

*denotes that the protective effect of polymorphism on detection or survival rate of individuals was different for different colours or colour morphs.
