# Supplementary material for: Colour Polymorphism Protects Prey Individuals and Populations Against Predation
Source: Sci Rep. 2016 Feb 23;6:22122. doi: 10.1038/srep22122 (PMC4763262; doi:10.1038/srep22122)
Supplement: Supplementary Information [file srep22122-s1.pdf]

Supplementary Information

Colour polymorphism protects prey individuals and populations against predation

Einat Karpestam, Sami Merilaita and Anders Forsman

Supplementary Text

Below is a brief summary of previous experimental studies that have been carried out in such a way that the results allow for evaluating the hypothesis put forward by Poulton [1,2] that polymorphism protects individuals and populations against predation.

Studies in which prey were presented sequentially. Pietrewicz and Kamil [3] presented five blue jays (*Cyanocitta cristata*) with series of slides showing images of *Catocala* moths photographed against their typical species substrate. They found that the birds improved their success rate of prey detection when the series consisted of a single prey species but not when the series consisted of a mixture of two different prey species.

Knill and Allen [4] used an experimental protocol in which human subjects were presented with artificial 'prey' displayed against complex backgrounds on computer screens in sequences that represented four levels (1, 2, 6 or 12 morphs) of polymorphism. They reported that detection rates of individual prey items decreased with increasing levels of polymorphism [4].

Karpestam, et al. [5] used a similar approach and asked human participants to search for images of natural black, striped or grey *Tetrix subulata* pygmy grasshopper morphs presented in sequence against photographs of their natural habitat on computer screens. Fewer grasshoppers were detected when morphs were presented in mixed than in uniform sequences, suggesting that natural levels of polymorphic variation can impede the efficiency of visually orientated predators and increase survival of individual prey [5].

Studies in which prey were presented in aggregations. In all the above studies prey were presented sequentially. The effects of polymorphism on predator efficiency and prey vulnerability may differ if predators instead encounter aggregations with multiple potential prey individuals. In many cases it would be realistic to hypothesize that subsequent encounters of prey would be connected to each other in time and place.

Croze [6] presented groups of painted mussel shells that concealed a piece of meat reward to carrion crows (*Corvus corone*) in the wild and found that individual mussel shells suffered more predation when presented in monomorphic populations than in trimorphic populations. However, a single pair of crows was used as predators in all replicates [6], and the results of that study should therefore be interpreted with caution.

Glanville and Allen [7] presented human subjects with 10 monomorphic and 10 extremely polymorphic (all individuals were different) populations of computer generated prey that resembled resting moths. In their study, the prey were presented in high or low density (20 or 4 prey presented simultaneously on a single screen), and the time until half of the prey had been detected was recorded. Subjects were faster at deleting half of the prey in their first monomorphic population than in their first polymorphic population, but they detected the prey equally fast in their last monomorphic as in their last polymorphic populations, and the level of polymorphism did not affect the number of mistakes made [7].

Studies in which prey were presented in aggregations to test for effects on survival of populations. In the studies described above, the protective polymorphism hypothesis was evaluated based on comparisons of fitness (rate of or time to detection) for individuals in polymorphic versus monomorphic treatments. We are aware of only one previous study that has tested whether polymorphism may lower predation in such way that it increases the survival of populations.

Wennersten and Forsman [8] used artificial prey made of dyed pastry in a field experiment and compared survival of polymorphic and monomorphic groups that were exposed to predation by wild birds. In this experiment coloration affected survival of both individuals and populations, but polymorphic populations that included prey of four different colours did not go extinct at a lower overall rate than monomorphic populations [8]. It is noteworthy that the colours of prey used in that experiment were not concealing; it has been hypothesized that the protective effect of polymorphism should be greater when prey are more difficult to detect [4,7,9].

Supplementary References

1 Poulton, E. B. *The colour of animals: their meaning and use.* (Kegan Paul, Trench, Trubner, 1890).

2 Poulton, E. B. Notes upon, or suggested by, the colours, markings and protective attitudes of certain lepidopterous larvae and pupae, and of a phytophagous hymenopterous larva. *Trans Entom Soc* **1884**, 27-60 (1884).

3 Pietrewicz, A. T. & Kamil, A. C. Search image formation in the blue jay (*Cyanocitta cristata*). *Science* **204**, 1332-1333 (1979).

4 Knill, R. & Allen, J. A. Does polymorphism protect? An experiment with human "predators". *Ethology* **99**, 127-138 (1995).

5 Karpestam, E., Merilaita, S. & Forsman, A. Natural levels of colour polymorphism reduce performance of visual predators searching for camouflaged prey. *Biol J Linn Soc* **112**, 546-555, doi:10.1111/bij.12276 (2014).

6 Croze, H. in *Searching Image in Carrion Crows* 58-72 (Paul Parey in Berlin und Hamburg, 1970).

7 Glanville, P. W. & Allen, J. A. Protective polymorphism in populations of computer-simulated moth-like prey. *Oikos* **80**, 565-571 (1997).

8 Wennersten, L. & Forsman, A. Does colour polymorphism enhance survival of prey populations? *Proc. R. Soc. B.* **276**, 2187-2194, doi: http://dx.doi.org/10.1098/rspb.2009.0252 (2009).

9 Merilaita, S. Frequency-dependent predation and maintenance of prey polymorphism. *J. Evol. Biol.* **19**, 2022-2030 (2006).
